# 

**Published:** 1878-10

**Authors:** 


### CONTENTS.

PAGE.

**CONTRIBUTIONS.**

Piano Plaints, Ben. H. Pratt ..................... 59
The Care of the Feet. P. Harold Hayes, M. D. ... 62
Always against the Woman. Ausburn Towner .... 64
A Man who did not Sweat. A. R. Kilpatrick, M. D. 67

**MEDICAL ITEMS AND NEWS.**

Freckles—Salicylic Mixture—Hair Tonic—Cod Liver Oil with Chloral—Bromide Eruption—Gargle—Cold Cream—Bichromate of Potash—Gout—Poison-Oak Eruption—Nævi—Salicylic Acid—Soporific—Anal Fissure—Cough Syrup—Castor Oil Mixture—Strangulated Hernia—Diarrhœa Mixture—Mothers' Marks—Wakefulness—Rheumatism—Whooping Cough—Sea-Sickness—Liniment—Rectal Alimentation—Pneumonia—Aural Discharges—Asthma—Boils—Danger from the Hypodermic Injection of Morphia—Ascites—Corns—Diseases of Ear from Bathing—Capsicum with Quinia—Whooping Cough ............... 69 to 75

**OUR CORRESPONDENCE.**

Eight Letters and Replies—A Communication on Quackery, ............................... 76 to 77

PAGE.

**HOUSEHOLD HINTS AND RECIPES.**

Orange Whey—Remedy for Rats—Shampoo-Fluid—Raised Waffles—Fruit Canning—Fly-Glue—Waterproof Paper—To Remove Ink—To Get Rid of Stumps—New Tanning Process—Painting Iron Work—Invisible Ink—Drying Oil—To Imitate Ground Glass—Large Filter--Hot Water for Plants ........................................ 78 to 79

**EDITORIAL.**

Yellow Fever ........................................ 80

Misapplied Sympathy ................................. 81

A Sulphur Bath ...................................... 81

Burns of the Eye .................................... 82

**EDITORIAL CHIT-CHAT.**

Hospitals and Hard Times—Ithaca—Preplexed Coroners—That Tent—Attractive—A Dangerous Nuisance—New Steamer—Well Answered—Lusus Naturæ—Beautiful Sculpture—The Races—State Fair—Power of Music—The Dobson—Flower Hunting ............................ 83 to 86

